# Molecular analysis of clinical *Citrobacter* spp. isolates: Acquisition of the *Yersinia* high-pathogenicity island mediated by ICE*kp* in *C. freundii*

**DOI:** 10.3389/fmicb.2023.1056790

**Published:** 2023-03-16

**Authors:** Guangcun Zhang, Qiang Zhao, Kun Ye, Liyan Ye, Yanning Ma, Jiyong Yang

**Affiliations:** Laboratory Medicine Department, First Medical Center of Chinese PLA General Hospital, PLA Medical College, Beijing, China

**Keywords:** *Citrobacter*, integrative conjugative element, yersiniabactin, high-pathogenicity island, *Citrobacter freundii*

## Abstract

**Background:**

Studies on *Citrobacter* spp. are limited, hindering our understanding of its species evolution and medical relevance.

**Methods:**

A total of 164 clinical *Citrobacter* spp. isolates were collected from 2017 to 2020 and identified by VITEK MALDI-TOF MS or VITEK-2 Gram-Negative Identification Card. All isolates were further analyzed by whole-genome sequencing using a HiSeq sequencer. All sequences were processed using different modules of the PGCGAP integrated package: Prokka and fastANI were used for annotation and average nucleotide identification (ANI), respectively. Antibiotic resistance and virulence genes were identified by searching CARD, ResFinder, and VFDB databases, respectively. Strains were identified using Ribosomal Multi-locus Sequence Typing (rMLST) classification based on 53 ribosome protein subunits (*rps*). The evolutionary relationship was analyzed using kSNP3 and visualized by iTOL editor v1_1. Genetic environments were compared by BLAST and visualized by Easyfig 2.2.5. The pathogenicity of some *Citrobacter freundii* isolates was confirmed by *Galleria mellonella* larvae infection test.

**Results:**

A total of 14 species of *Citrobacter* spp. were identified from 164 isolates. However, 27 and 11 isolates were incorrectly identified as *C. freundii* and *Citrobacter braakii* by MALDI-TOF MS, respectively. In addition, MS also failed to identify *Citrobacter portucalensis*. The virulence genes mainly encoded proteins related to flagella and iron uptake systems. *Citrobacter koseri* isolates (*n* = 28) contained two iron uptake systems, coding yersiniabactin and aerobactin, respectively. *C. braakii* isolates (*n* = 32), like *Salmonella*, carried Vi capsule polysaccharide synthesis genes. The yersiniabactin gene clusters identified in five *C. freundii* isolates are located on various ICE*kp* elements and have not been reported previously. Moreover, ICE*kp*-carrying *C. freundii* showed diverse pathogenic features.

**Conclusion:**

Conventional methods have significant defects in identifying *Citrobacter* spp. ICE*kp*-like elements-mediated acquirement of the *Yersinia* high-pathogenicity island was identified for the first time in *C. freundii*.

## Introduction

*Citrobacter* spp. are facultatively anaerobic, Gram-negative *Enterobacterales* bacteria. They usually colonized soils, waters, and animal or human intestines (Yuan et al., 2019b). Although *Citrobacter* spp. have yet to be considered important hospital-associated pathogens, they are related to hospitals and community-acquired infections of the respiratory tract, urinary tract, bloodstream, and central nervous system, especially in immunocompromised populations (Liu L. H. et al., 2018; Qian et al., 2018).

To date, 18 species have been named in the genus *Citrobacter* (Wang et al., 2021). *Citrobacter* spp. are often misidentified by traditional identification methods (Hasan et al., 2019; Oberhettinger et al., 2020), hindering our understanding of their evolution and medical relevance. With the development of whole-genome sequencing (WGS), *Citrobacter* spp. species can be distinguished more precisely, which advances research on their species identification, evolutionary relationships, and genome structures (Yap et al., 2020; Dziri et al., 2021). However, most studies on *Citrobacter* spp. have focused on the common species *Citrobacter koseri* and *Citrobacter freundii* and their phenotypic multi-drug resistance, pathogenicity, and related mechanisms (Liu L. et al., 2018; Yuan et al., 2019a; Liu et al., 2021).

Multiple siderophore systems, including enterobactin, yersiniabactin, salmochelin, and aerobactin in the family *Enterobacteriaceae*, have been associated with greater pathogenic potentials (Kramer et al., 2020). The *Yersinia* high-pathogenicity island (HPI) is widely distributed among different members of the family *Enterobacteriaceae* (Bach et al., 2000). The role of HPI is to synthesize iron carriers through gene clusters in an iron-deficient environment, compete for iron binding in their environment, form a complex that is recognized by recognition sites on the cell surface, and enter cell for bacterial use (Kramer et al., 2020).

Mobile genetic elements (MGEs) play a role in resistance genes’ and virulence factors’ capture, accumulation and transmission. They mainly include insertion sequences, transposons, gene cassettes/integrons, plasmids, and integrative conjugative elements (ICEs) (Partridge et al., 2018). The ICEs are self-transmissible MGEs that can be transmitted and expressed in different bacteria, allowing them to acquire different genotypes and phenotypes, facilitating their adaptation to environmental changes, and increasing their pathogenicity (Johnson and Grossman, 2015).

There is an HPI-associated ICE in *Enterobacteriaceae*, mainly in *Klebsiella pneumoniae* and *Escherichia coli*, named ICE*kp* (Lin et al., 2008). The ICE*kp* consists of a 5′ end, which contains a P4-like *int* and an HPI, a type IV secretion system (T4SS), and a 3′ end which is associated with DNA conjugative transfer. ICE*kp* is mainly distributed in *Klebsiella* spp., while ICE*kp*-like elements are also present in *E. coli*, *C. koseri*, *Yersinia pestis*, *Yersinia pseudotuberculosis*, and *Enterobacter hormaechei*. There are many subfamilies in the ICE*kp* family (Lam et al., 2018). Among them, ICE*kp1* family contains fourteen members (ICE*kp*1.1–1.14), and yersiniabactin-encoding genes (*ybt*) contain 17 members (*ybt*1–*ybt*17) (Lin et al., 2008; Lam et al., 2018). Recent studies have identified ICE*kp2* family, which is completely different from the ICE*kp1* family but also possesses modules mediating the movement of elements and other accessory genes (Farzand et al., 2019). Although ICE*kp2* family cannot mediate its own transmission between bacteriophages, it enhances the transmission of ICE*kp1* family when coexisting (Farzand et al., 2019). Similar phenomenon also exists between ICE*Th1* and ICE*Th2* in *Thermus thermophilus* (Baquedano et al., 2020).

*Citrobacter koseri* is the only *Citrobacter* spp. that has been reported to contain an HPI (Yuan et al., 2019b). Its HPI is mainly related to the HPI among *Y. pestis* and is located on the chromosomes for conserved clonal transmission and pathogenicity enhancement (Yuan et al., 2019b). In addition, *C. koseri* also possesses HPI-related ICE*kp1* family with unverified pathogenicity (Lam et al., 2018). In this study, for the first time, we identified ICE-mediated HPI transmission in *C. freundii*. We also found that ICE might spread across strains and even genera to allow the same genotype or phenotype to cross. Furthermore, we also investigated the evolutionary relationships of *Citrobacter* spp. through genome-wide sequence comparisons for species identification.

## Materials and methods

### Bacterial strains and antimicrobial susceptibility testing

A total of 164 clinical *Citrobacter* spp. isolates were collected from our hospital from 2017 to 2020. The species of the isolates was first identified by using VITEK MALDI-TOF MS. The isolates were spotted on the target plate of VITEK MALDI-TOF MS, and then 1 μl VITEK MS-CHCA matrix was applied over the sample until the matrix and sample were dried and co-crystallized. The target plate with all prepared samples then was loaded into the VITEK MS system to acquire the mass spectra of whole bacterial cell protein. Based on the characteristics of ribosomal protein, the species of the isolates were determined by the comparison of the known mass spectra contained in the database. Moreover, we confirmed the species and tested the antimicrobial susceptibility of the isolates by biochemical assays with the VITEK 2 Compact system. The isolates were adjusted to the concentration of McFarland standard 0.5∼0.63. In 3.0 ml sodium chloride solution (0.45%), and then 145 μl bacterial solution was taken into 3.0 ml phosphate buffer solution with the insertion of VITEK-2 Gram-Negative Identification Card and AST-GN13 card for the bacterial identification and antimicrobial susceptibility, respectively. AST-GN13 card contains cephalosporins, carbapenems, aminoglycosides, quinolones, and other antibiotics. The results were interpreted with reference to CLSI M100. *E. coli* 8739 and 25922 were used as reference strains.

### Whole-genome sequencing and data analysis

All isolates were subjected to WGS using a paired-end library with an average insert size of 350 bp (ranging from 150 to 600 bp) on a HiSeq sequencer (Illumina, CA, USA). All sequences were processed using different modules of the PGCGAP integrated package. Data were assembled using SPAdes, annotated using Prokka and subjected to fastANI for whole-genome ANI. ANI-related heatmaps were generated using self-developed scripts “triangle2list.pl,” “get_ANImatrix.pl” and “Plot_ANIheatmap.R.” Resistance genes and virulence genes were identified using CARD, ResFinder, and VFDB databases, respectively. Strains were identified using the Ribosomal Multi-locus Sequence Typing (rMLST) classification based on 53 ribosome protein subunits (*rps*) available on the PubMLST website. The evolutionary relationship was analyzed using kSNP3 and visualized by iTOL editor v5. Genetic environments were compared by BLAST and visualized by Easyfig 2.2.5. The bacterial genome data has been uploaded to NCBI under BioProject accession PRJNA885261.

### *Galleria mellonella* larvae infection test

Fresh greater wax moth larvae were used for virulence testing as previously described (Gu et al., 2018). Overnight *C*. *freundii* cultures were suspended in phosphate-buffered solution at a concentration of 1.5 × 10^8^ CFU/ml. After injecting about 10 μl of the suspension into the ventral cavity above the anterior left hind leg, the larvae were placed in a 37°C incubator to observe their survival. Ten injections of each strain were made, and the test was repeated three times.

## Results

### Phenotypic characteristics and species identification of *Citrobacter* spp. isolates

All 164 *Citrobacter* spp. isolates were collected from clinical specimens, including urine (*n* = 92, 56.10%), bile (*n* = 49, 29.88%), and blood (*n* = 23, 14.02%). Analysis of sequencing data (rMLST and FastANI), revealed that 27 and 11 isolates were incorrectly identified as *C. freund*ii and *Citrobacter braakii* by MALDI-TOF MS, respectively. In addition, MS failed to identify *Citrobacter portucalensis* (Table 1). The majority of *Citrobacter* spp. isolates exhibited higher resistance to the third-generation cephalosporins ceftazidime and ceftriaxone and were susceptible to other clinically commonly used antibiotics (Table 1). Several *C. freundii* and *C*. *portucalensis* isolates were resistant to carbapenems, and most of them carried *bla*_*NDM–*1_ or *bla*_*NDM–*5_ (Supplementary Table 1). *C*. *koseri* was highly susceptible to all types of antibiotics, possibly because it contained fewer resistance genes (Supplementary Table 1).

**TABLE 1:** The identification and drug resistance rate of *Citrobacter* spp.

Species	Identification methods			Drug resistance rate (%)							
	MS	rMLST	FastANI	CRO	CAZ	FEP	IPM	ETP	CIP	LEV	AK
*C. amalonaticus*	2	2	2								
*C*. *braakii*	43	32	32	65.62	56.25	9.38	0	0	37.5	18.75	3.13
*C*. *cronae*	0	3	3								
*C*. *europaeus*	0	5	5								
*C*. *farmeri*	1	0	0								
*C*. *freundii*	72	45	45	37.78	28.89	6.67	4.44	4.44	40	22.22	0
*C*. *koseri*	28	28	28	7.14	3.57	3.57	0	0	0	0	0
*C*. *murliniae*	0	2	2								
*C*. *pasteurii*	0	1	1								
*C*. *portucalensis*	0	35	36	47.22	33.33	8.33	8.33	11.11	41.67	27.78	0
*C*. *sedlakii*	1	1	1								
*C*. *telavivensis*	0	1	1								
*C. werkmanii*	6	0	0								
*C*. *youngae*	6	5	5								
Unidentified species	5	4	3								

CRO, ceftriaxone; CAZ, ceftazidime; FEP, cefepime; IPM, imipenem; ETP, ertapenem; CIP, ciprofloxacin; LEV, levofloxacin; AK, amikacin.

### Phylogenetic analysis

**FIGURE 1:**
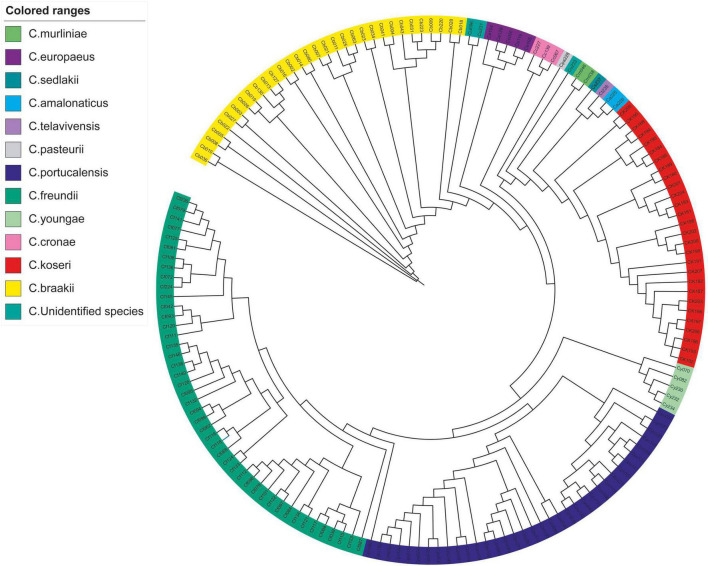
Whole-genome SNP phylogeny of *Citrobacter* spp. A phylogenetic tree of 164 *Citrobacter* spp. isolates was constructed based on whole-genome SNP using kSNP3.

**FIGURE 2:**
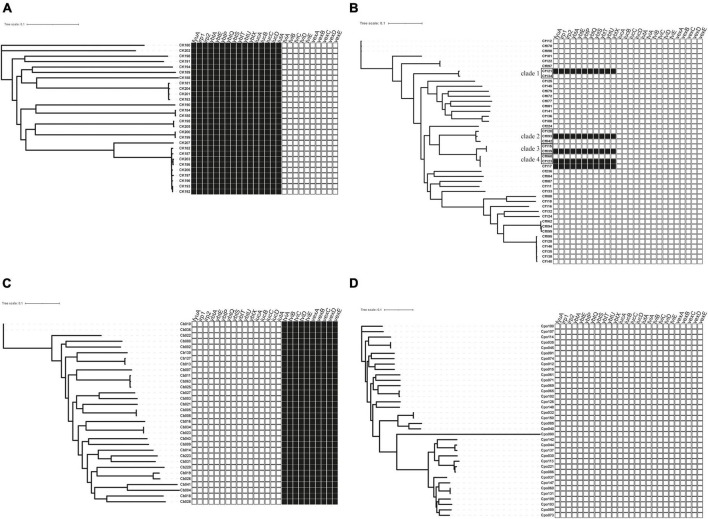
The distribution of virulence factors across *Citrobacter* spp. Boxes represent iron uptake and Vi capsule virulence factors derived from whole-genome data comparison with VFDB. **(A)** *C*. *koseri*, **(B)** *C*. *freundii*, **(C)** *C*. *braakii*, and **(D)** *C*. *portucalensis*. 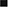, exist; 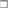, absence. For a full outline of sources, see Supplementary Table 1.

The *k*-mer based analysis of whole-genome SNPs revealed that strains belonging to the same species showed higher genetic relationships (Figure 1). The isolate Cpo90 was distant from other *C. portucalensis* and more closely related to *C. freundii* clade. Individual evolutionary trees of each species showed that *C. koseri* and *C. freundii* had small outbreaks, while the other strains were more dispersed (Figure 2).

### Prevalence of virulence genes

The virulence genes mainly encoded flagella and iron uptake-related systems. *C. koseri* contained two iron uptake systems, coding yersiniabactin and aerobactin (Figure 2A). The complete genomes and GenBank annotations of 11 *C. koseri* strains were downloaded from NCBI. These genomes possessed multiple copies of structure-uncorrelated genes encoding yersiniabactin and aerobactin. A comparison of our sequencing data with the above complete genomes using BLAST showed similar molecular characteristics (data not shown). *C. braakii*, like *Salmonella*, carried Vi capsule polysaccharide synthesis genes (Figure 2C), while *C. portucalensis* contained the lowest number of virulence genes (Figure 2D). Surprisingly, we, for the first time, identified the yersiniabactin gene cluster in five *C. freundii* isolates (Figure 2B).

### ICE*kp*-mediated acquisition of high pathogenic island in *C. freundii*

**FIGURE 3:**
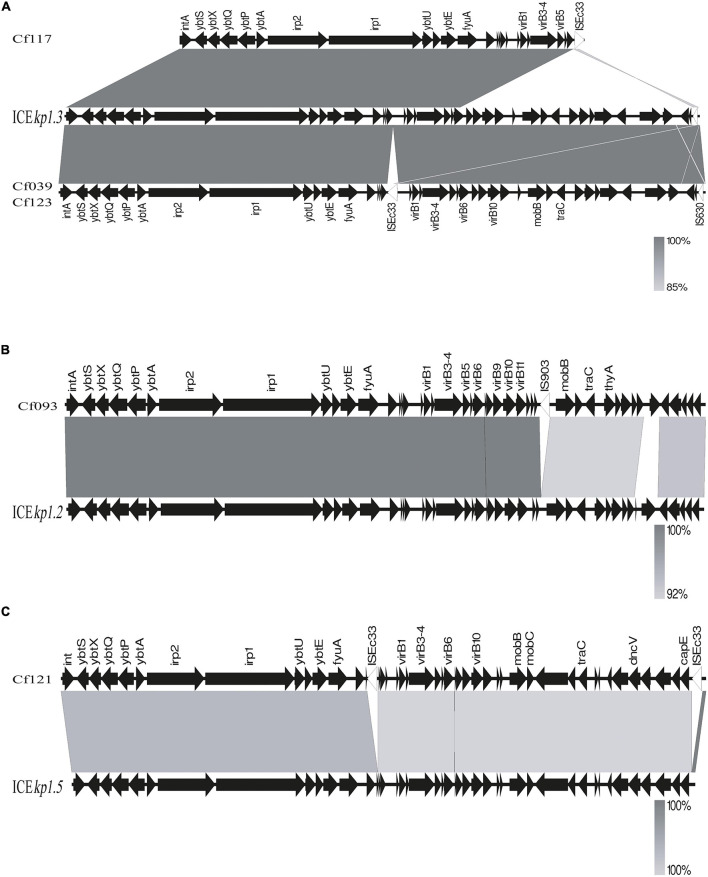
Linear comparison of ICE*kp* sequences in five *C*. *freundii*. Sequences were aligned to one another using Easyfig. Genes are denoted by arrows with a gradient from gray (low identity) to black (high identity). **(A)** Genomic comparison of Cf117, Cf039, Cf123 with ICE*kp*1.3, **(B)** Cf093 with ICE*kp*1.2 and **(C)** Cf121 with ICE*kp*1.5.

BLAST and annotation analysis revealed that all *C. koseri* isolates carried a cluster of yersiniabactin genes as conserved elements. However, the *Yersinia* HPI in four *C. freundii* isolates was located on various ICE*kp* (Figure 3). The HPI structure of Cf039 and Cf123 was highly homologous to ICE*kp1.3*, which has an insertion of IS630 family transposase ISEc33 in the putative functional region between HPI and T4SS secretion system (Figure 3A). Genetic structure of Cf093 was similar to that of ICE*kp1.2*, which has an insertion of IS*5* family transposase IS*903* before *mobB* (Figure 3B). The HPI structure of Cf121 had an IS*Ec33* inserted between *ybt* and T4SS of ICE*kp1.5* (Figure 3C). Interestingly, Cf117 carried only partial ICE*kp1.3* sequence, which might result in functional defects (Figure 3A).

### Comparison of the pathogenicity of *C. freundii*

**FIGURE 4:**
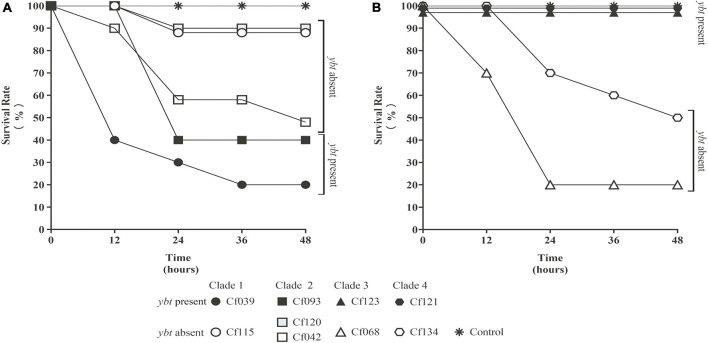
The survival rate of *Galleria mellonella* larvae. The solid black symbol represents *C. freundii* carrying *ybt*. The hollow white and gray symbol represent *ybt*-negative strains. The same shape represents the strains within the same clade. The values were fine-tuned to differentiate the folds with the same survival rate. All results were rounded to the nearest integer values. Strains with *ybt* had a lower **(A)** or higher **(B)** survival rate.

Several *C. freundii* isolates with HPI were used to infect *Galleria mellonella* larvae. Meanwhile, HPI-negative *C. freundii* isolates of the same clade (Figure 2B) were used as controls. The survival rates of HPI-carrying Cf039 and Cf093 were 20 and 40%, respectively. The survival rates of Cf123 and Cf121 were 100% at 48 h after infection (Figure 4). The survival rates of five HPI-negative strains Cf134, Cf042, Cf115, Cf068, and Cf120 were 50, 50, 90, 20, and 90%, respectively (Figure 4).

## Discussion

In this study, 164 clinical *Citrobacter* spp. isolates were identified by MALDI-TOF MS, rMLST and ANI. rMLST and ANI can identify most *Citrobacter* species more accurately, while *C. portucalensis* were incorrectly identified as *C. freundii* by MALDI-TOF MS (Table 1). Previous studies have also shown that MALDI-TOF MS has a lower identification accuracy and can misidentify intraspecies such as *C. portucalensis* and *C. freundii*, which are generally considered the most prevalent *Citrobacter* species in the clinics (Rödel et al., 2019). Since MS is the main identification method routinely used in most clinical laboratories, and rMLST and ANI are difficult to carry out, the low identification accuracy of MS may lead to large errors in the epidemiological studies of *Citrobacter* spp. and may interfere with drug selection in empirical anti-infection therapies. However, *Citrobacter farmeri* and *Citrobacter werkmanii* are exceptions because they can be accurately identified by MALDI-TOF MS, but not by rMLST and ANI (Table 1), indicating that different identification methods have different accuracy for different species. Therefore, supplementary methods, such as digital DNA-DNA hybridization computation and biochemical characterization are needed for identifying some species (Oberhettinger et al., 2020).

Phylogenetic analysis has shown that clinical *Citrobacter* spp. exhibits high level of clonal diversity. The majority of strains exhibit interspecies differences (Figure 1). The isolates of the main species also show intraspecific differences (Figure 2), while only *C. koseri* (Figure 2A) and *C. freundii* (Figure 2B) caused small-scale outbreaks. Several *C. freundii* and *C. portucalensis* isolates exhibit resistance to carbapenems due to the acquisition of various types of *bla*_*NDM*_. This suggests we should pay attention to the risk of transmission caused by some species, such as *C. portucalensis*, which can acquire foreign resistance genes (Cao et al., 2021).

Most *Citrobacter* spp. carry various virulence factors related to flagella apparatus biosynthesis and iron uptake system (Figure 2). *C. koseri* contains the most virulence factors and uniquely possesses two groups of iron uptake systems (yersiniabactin and aerobactin). HPI is an important factor closely related to the pathogenicity of *C. koseri* (Yuan et al., 2019b). We have revealed that *ybt* carried by *C. koseri* is rarely present in ICE*kp*, which is mainly carried by various ICE*kp* in other *Enterobacteriaceae* (Lam et al., 2018). In addition, most *C. koseri* isolates also contain aerobactin. However, analysis of their complete sequence revealed that the genes coding aerobactin and yersiniabactin are not genetically linked, and the biological effects of carrying multiple siderophores need to be further confirmed. Interestingly, *C. koseri* isolates carry fewer drug resistance genes (Supplementary Table 1) and exhibited lower drug resistance than other species (Table 1). Conversely, *C. portucalensis* contains fewer virulence factors (Figure 2D) and relatively more drug resistance genes (Supplementary Table 1). It has been suggested that high virulence and high resistance populations do not overlap. However, the possibility of dual expression of high virulence and high resistance is increasing through genomic changes (Bialek-Davenet et al., 2014). In addition, all *C. braakii* isolates contain clusters of genes associated with Vi capsular polysaccharide, (Hu et al., 2017; Qian et al., 2018; Yuan et al., 2019b).

*Citrobacter freundii* can produce large amounts of Vi and attach to the cell surface, and the *viaB* locus can affect changes in Vi antigen expression (Snellings et al., 1981). However, our results showed that *C. freundii* does not contain any Vi capsular synthesis gene cluster (Figure 2B), suggesting that the Vi cluster may be externally obtained but not transmitted by a conserved clone.

Through BLAST analysis, we found that five strains of *C. freundii* in this study contained yersiniabactin gene cluster (HPI) (Figure 2), and seven of all *C. freundii* sequences downloaded on NCBI also contained HPI. The genomic comparative analysis found that all but one HPIs are present in various ICE*kp* of ICE*kp1* family with high similarity (Figure 3; Lam et al., 2018). This is the first report on ICE*kp*-mediated HPI among *C. freundii*. The ICE*kp* differs from the hosts in genomic GC content, suggesting that these HPIs may be transmitted through ICE level. *C. freundii* is the most prevalent *Citrobacter* spp. with the most carbapenem-resistant genes (Arana et al., 2017). Once multi-drug resistant *C. freundii* acquires HPI and spreads widely, it will make treatment more difficult. However, our results showed that clinical *C. freundii* isolates with HPI do not exhibit higher pathogenicity than HPI-negative isolates (Figure 4). The possible relevant mechanisms include (1) ICE may not exhibit its functions when bound to the host’s chromosome and only functions after it has been sheared down to form a loop (Johnson and Grossman, 2015) and (2) HPI-containing strains activate host’s autophagy, making the HPI-carrying host at a disadvantage stage at certain point (Dalmasso et al., 2021). From the perspective of virulence factors contained in the whole, through comparison of VFDB database, it was found that clade3 and clade4 had no difference in virulence factor except HPI gene cluster. Except that clade1 and clade2 contain different HPI gene clusters, Cf039 in clade1 contains gene clusters encoding fimbriae while Cf115 does not. The results in clade2 are opposite (Figure 4A). Fimbriae are important factors for bacteria to adhere to host cells and play a crucial role in their colonization on the host. However, according to the results of this study, it may be considered that the pathogenicity of fimbriae on bacteria needs further study.

Our study has several limitations. First, *Citrobacter* spp. were collected from single hospital. Second, the transmission ability of ICE*kp* in *C. freundii*, and whether the insertion of IS affects the propagation of ICE*kp* were not analyzed.

## Data availability statement

The datasets presented in this study can be found in online repositories. The names of the repository/repositories and accession number(s) can be found below: https://www.ncbi.nlm.nih.gov/genbank/, PRJNA885261.

## Author contributions

JY designed the study. GZ, QZ, KY, LY, and YM did phenotypic and genotypic analysis. GZ and JY drafted the manuscript. All authors read and approved the final manuscript.
